# What’s hot and what’s not: Making sense of biodiversity ‘hotspots’

**DOI:** 10.1111/gcb.15443

**Published:** 2020-11-26

**Authors:** Murray S. A. Thompson, Elena Couce, Thomas J. Webb, Miriam Grace, Keith M. Cooper, Michaela Schratzberger

**Affiliations:** ^1^ Centre for Environment, Fisheries and Aquaculture Science (CEFAS), Lowestoft Laboratory Lowestoft Suffolk UK; ^2^ Department of Animal & Plant Sciences University of Sheffield Sheffield UK

**Keywords:** biodiversity, biodiversity hotspot, conservation, diversity partitioning, marine benthic fauna, Random Forest analysis, rarefaction and extrapolation, species richness

## Abstract

Conserving biogeographic regions with especially high biodiversity, known as biodiversity ‘hotspots’, is intuitive because finite resources can be focussed towards manageable units. Yet, biodiversity, environmental conditions and their relationship are more complex with multidimensional properties. Assessments which ignore this risk failing to detect change, identify its direction or gauge the scale of appropriate intervention. Conflicting concepts which assume assemblages as either sharply delineated communities or loosely collected species have also hampered progress in the way we assess and conserve biodiversity. We focus on the marine benthos where delineating manageable areas for conservation is an attractive prospect because it holds most marine species and constitutes the largest single ecosystem on earth by area. Using two large UK marine benthic faunal datasets, we present a spatially gridded data sampling design to account for survey effects which would otherwise be the principal drivers of diversity estimates. We then assess γ‐diversity (regional richness) with diversity partitioned between α (local richness) and β (dissimilarity), and their change in relation to covariates to test whether defining and conserving biodiversity hotspots is an effective conservation strategy in light of the prevailing forces structuring those assemblages. α‐, β‐ and γ‐diversity hotspots were largely inconsistent with each metric relating uniquely to the covariates, and loosely collected species generally prevailed with relatively few distinct assemblages. Hotspots could therefore be an unreliable means to direct conservation efforts if based on only a component part of diversity. When assessed alongside environmental gradients, α‐, β‐ and γ‐diversity provide a multidimensional but still intuitive perspective of biodiversity change that can direct conservation towards key drivers and the appropriate scale for intervention. Our study also highlights possible temporal declines in species richness over 30 years and thus the need for future integrated monitoring to reveal the causal drivers of biodiversity change.

## Graphical abstract

Conserving biodiversity ‘hotspots’ is intuitive because finite resources can be focussed towards manageable units. Yet, biodiversity, environmental conditions and their relationship are more complex with multidimensional properties. Assessments which ignore this risk failing to detect change, identify its direction, or gauge the scale of appropriate intervention. We focus on the marine benthos which holds most marine species and constitutes the largest single ecosystem on earth by area. When assessed alongside environmental gradients, α‐, β‐ and γ‐diversity provide a multidimensional but still intuitive perspective of biodiversity change that can direct conservation towards key drivers and the appropriate scale for intervention. Photographs used are a combination of © Elena Couce and © Crown Copyright, the latter collected by Cefas, Cornwall IFCA and Environment Agency whilst undertaking survey work in partnerships with the JNCC and Natural England.

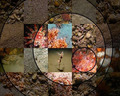

## 1 INTRODUCTION

In light of the global biodiversity crisis (Butchart et al., 2010; Johnson et al., 2017; Loh et al., 2005; McRae et al., 2017), robust information on the spatial distribution of biodiversity and anthropogenic drivers of change are critical to direct conservation efforts towards the most effective conservation interventions (Edgar et al., 2016; McGill et al., 2015; Pimm et al., 2014). Prioritizing ‘hotspots’ with high species richness or distinct assemblages is one of the most common strategies for biodiversity conservation (Norman & White, 2019). Yet, there are many limitations in the way biodiversity estimates are generated because of how data are collected, collated and analysed (Cardinale et al., 2018; Engemann et al., 2015; Guilhaumon et al., 2008; Kupschus et al., 2016), which hampers biodiversity conservation (McGill et al., 2015; Pimm et al., 2014).

Different views exist about how assemblages are structured. The ‘superorganism’ view predicts that strong species interactions cause sharp boundaries between unique assemblages (Clements, 1916). This implies that monitoring and conservation can target distinct assemblages. The ‘individualistic’ view predicts species competitiveness is conserved irrespective of composition (Gleason, 1926; akin to neutral theory; Hubbell, 2001); hence, compositional changes can be largely explained by species dispersal and responses to environmental gradients which can be both gradual and discontinuous (Liautaud et al., 2019). This view implies that, where there is little environmental change, stochastic processes will largely determine local assemblage structure and conservation measures can be targeted at specific pressures affecting species across their distributional range. The superorganism view is implicit in maps with hard edges between adjacent habitats and is widely applied to inform biodiversity conservation and monitoring strategies (e.g. EUNIS; Andersen et al., 2018; Coltman et al., 2008), although such classifications can perform poorly when related to changes in community composition (Cooper et al., 2019). This highlights a current and critical disconnect between how assemblages are structured and how conservation is applied. There is also compelling evidence that neutral processes alone are not adequate to describe community structure (Connolly et al., 2014; McGill, 2003). These competing views likely represent continuum extremes which are also not mutually exclusive in that a strongly interacting community could coexist with more loosely assembled species (Liautaud et al., 2019). The often right‐skewed distribution of interaction strengths in many food webs supports this (i.e. many weak and few strong, e.g. Bascompte et al., 2005; Emmerson & Raffaelli, 2004; Paine, 1992; Thorson & Barnett, 2017; Vázquez et al., 2007; Wootton, 1997), as does work by Vergnon et al. (2009) which showed niche and neutral processes were both important structuring forces on a single assemblage. Are we therefore dealing with (a) definable communities each of which respond to environmental change as a unit, (b) assemblage continua where species respond independently from one another, or both and how best should these be assessed and conserved?

Challenges associated with large‐scale biodiversity assessments have led to the proliferation of so‐called ‘big data’ (Edgar et al., 2016), such as online databases documenting taxonomy (e.g. the World Register of Marine Species; WoRMS Editorial Board, 2017), species distributions (e.g. MacroBen; Vanden Berghe et al., 2009; Global Biodiversity Information Facility; http://www.gbif.org/; Ocean Biogeographic Information System; OBIS, 2020), time series (e.g. BioTIME; Dornelas et al., 2018), and their relation to large‐scale environmental information (e.g. the Copernicus Marine Environment Monitoring Service; http://marine.copernicus.eu). There is much less data at similar scales, however, on species interactions or dispersal, which play critical roles in structuring assemblages (Albouy et al., 2019; Bradbury et al., 2008). Furthermore, data relevant for estimating species distributions are often derived from museum collections or from surveys conducted for other reasons, including establishing protected areas or environmental impact assessment (Cooper & Barry, 2017; Engemann et al., 2015; Norman & White, 2019). As such, they may not be best suited to providing evidence of the underlying reasons for observed changes at large scales (Arkema et al., 2006; Dickey‐Collas, 2014; Kupschus et al., 2016). Such data also contain many other potential issues that could compromise our ability to detect and understand changes in biodiversity, including spatiotemporal variation in abundance (sample size), sampling effort, a mixture of taxonomic resolutions, and the incorporation of both count and incidence (i.e. presence only) data (Cardinale et al., 2018; Engemann et al., 2015; Norman & White, 2019). Consequently, large‐scale biodiversity assessments have often relied on species distributions and species area relationships to define biodiversity hotspots (Elith & Leathwick, 2009; Norman & White, 2019), while lacking information on interactions, dispersal, temporal change, variation in the effect of area on biodiversity or sampling artefacts that could confound such predictions (Araújo & Guisan, 2006; Gilman et al., 2010; Guilhaumon et al., 2008; Liautaud et al., 2019).

Joint use of γ‐diversity (regional richness) with diversity partitioned between α (local richness) and β (dissimilarity) components offers a potential means to navigate these issues. Diversity partitioning is well established in the wider ecological literature (Chao et al., 2012; Hautmann, 2014; Jost, 2007; Na & Kiessling, 2015; Olszewski, 2004; Whittaker, 1960, 1972) but has not yet been incorporated into conservation programmes to assess status or inform where pressures on biodiversity could be mitigated. Yet, their incorporation could reveal the relative importance of local to large‐scale processes determining biodiversity patterns and thus whether it makes sense to clearly delineate a hotspot from other areas. Differences in response can also be indicative of how assemblage structure is changing, for example, large decreases in β‐diversity but not γ‐diversity would indicate species composition was homogenizing but that overall richness was unaffected (Dornelas et al., 2014; Magurran et al., 2018; McGill et al., 2015). Rarefaction and extrapolation with Hill numbers provide a unified framework within which to estimate these diversity metrics that can account for the sampling biases associated with variation in abundance and sampling effort, and can also be applied to many data types (Chao et al., 2014; Jost, 2007; Leinster & Cobbold, 2012).

Marine assemblages are unique in that they lack some of the dispersal constraints experienced by organisms in terrestrial ecosystems (Baselga et al., 2012; Jones, 2016; Kay & Palumbi, 1987; Kinlan & Gaines, 2003). They are nevertheless experiencing comparable rapid and profound defaunation with widespread impacts on biodiversity (Blowes et al., 2019; Dornelas et al., 2014; McCauley et al., 2015; Pimm et al., 2014; Webb & Mindel, 2015). Much of the marine species biodiversity is found in the benthos which constitutes the largest single ecosystem on earth by area (Snelgrove, 1999; Snelgrove et al., 1997). The ability to clearly delineate marine benthic communities is an attractive prospect from an operational perspective since large areas could be categorized into manageable sampling units (e.g. Cooper et al., 2019). However, without evidence of sharp changes in assemblage structure, monitoring and conservation strategies which assume marine ecosystems can be compartmentalized remain contentious.

Much work has been done to establish the drivers or at least environmental correlates of marine benthic faunal diversity patterns. Depth, sediment composition, various measures of water velocity or wave action and proximity to coast have all been shown to be useful descriptors (Barrio Froján et al., 2012; Duineveld et al., 1991; Rees et al., 1999; Renaud et al., 2009). Somerfield et al. (2009) showed that, rather than competition for resources which would be defined locally and favour assemblages composed of species less similar to one another, benthic assemblages tended to contain species more closely related to each other than would be expected by chance, and thus were likely organized primarily by large‐scale environmental processes. However, it was not clear if the diversity of the most abundant taxa, polychaetes, was independent of, or driven by both, local and large‐scale processes. Renaud et al. (2009) showed that local species richness was positively correlated with large‐scale richness. From a conservation perspective, it would be useful therefore to know whether local‐scale, large‐scale processes or some combination of these drive high biodiversity, and whether high local diversity is a reliable measure of high diversity at larger geographical scales.

To address these evidence needs, we draw together data from across the UK Exclusive Economic Zone (UK EEZ), and across many different habitats (e.g. Andersen et al., 2018; Coltman et al., 2008; Cooper et al., 2019), where extensive spatial and temporal observations exist for benthic biota and prevailing environmental conditions. We focus on two large faunal datasets and observations between 1985 and 2016 where complementary covariate data exist: the first was compiled for a UK benthic macrofaunal distribution study (henceforth 'BM data'; Cooper & Barry, 2017) containing count data for 1,964 species with 23,153 multi‐species observations (i.e. those collected at the same time and at the same location); the second was downloaded from the largest publicly available online database documenting the global distribution of marine species, Ocean Biogeographic Information System (OBIS, 2020), which contained 3,187 benthic species with a mixture of count and incidence data with 23,646 multi‐species observations (henceforth 'OBIS data’). To the authors’ knowledge, the combined dataset represents the largest used to assess UK marine benthic diversity with a total of 3,626 benthic species from 46,799 multi‐species observations.

After accounting for survey effects, we estimate biodiversity across a range of scales to test the following hypotheses: (a) benthic biodiversity hotspots are inconsistent across metrics; (b) α‐, β‐ and γ‐diversity relate uniquely to covariates; and (c) both sharply defined ‘superorganism’ and loosely collected ‘individualistic’ communities exist along a β‐diversity continuum. We aimed to explore whether robust biodiversity hotspots can be clearly delineated and reveal which environmental drivers explain the most variation in α‐, β‐ and γ‐diversity. At the same time, this approach allowed us to test the limitations of using aggregated biodiversity data in this context. Taken together, this information will be valuable in highlighting current knowledge gaps, chief drivers of biodiversity change and the appropriate scales for assessment and conservation to operate.

## 2 MATERIALS AND METHODS

### 2.1 Benthic data collation

We used benthic macrofaunal (BM) abundance data collated by Cooper and Barry (2017; https://doi.org/10.14466/CefasDataHub.34), consisting of 33,198 grab samples from 777 surveys collected between 1969 and 2016 from UK waters, sourced from both government and industry, including contributions from the aggregate dredging and energy sectors. Samples taken from known impacted sites, for example, within actively dredged locations, and species known to be reported inconsistently, for example, colonists or fish, were excluded. The data were collected with a variety of grab‐types, with 93% coming from either a 0.1 m^2^ Hamon grab or a 0.1 m^2^ Day grab, with the majority of surveys conducted post 2000 and between June and September. See Cooper and Barry (2017) for a detailed description of the complete dataset.

Benthic survey data were also obtained from the Ocean Biogeographic Information System (Grassle, 2000; OBIS, 2020), the largest online free‐access global marine database of species records. The data used in this study were downloaded on 19/07/2017 and can be accessed at: https://doi.org/10.25607/obis.export.dda4657a. These data include incidence and, in some cases, abundance information for marine taxa within the UK EEZ between 1750 and 2014, with the majority of observations between 1980 and 2012. There were insufficient reported observations to assess biodiversity across spatial scales post‐2012, so these entries were removed. Taxonomic information was standardized and identified as benthic or otherwise using the World Register of Marine Species online Taxon Match Tool and Functional Group classifications, respectively (WoRMS; www.marinespecies.org). WoRMS has aggregated data on species attributes including broad ‘functional groups’, which we use to identify benthic faunal taxa. We do this using a dedicated R function (https://github.com/tomjwebb/WoRMS‐functional‐groups) which accesses the WoRMS API using the *worrms* R package (Chamberlain, 2019). Our ‘benthos’ group includes all species categorized in WoRMS as endobenthos, epibenthos, hyperbenthos, macrobenthos, meiobenthos and microbenthos, as well as those originally classified simply as benthos. When separate functional groups are recorded for different life stages, we always use the group for the adult stage. The resulting set of benthic taxa were broadly comparable across OBIS and BM data based on phylum occurrences (Table S1). We retained benthic occurrences contained in OBIS data regardless of survey methodology, meaning that our final dataset included records from a range of methods including benthic grabs, trawls and dredges. Therefore, OBIS data were standardized to incidence for meaningful comparison. Multi‐species observations with low numbers of observations and/or species could be indicative of a survey targeting specific species, while not recording others. Those with fewer than 30 observations and/or 10 species were therefore not considered. OBIS data included in this study consisted of 3,187 benthic species from 23,646 multi‐species observations.

Drawing on diverse data sources is typical to biodiversity hotspot assessment (Norman & White, 2019). This is because it is essential to aggregate observations from across habitats or species that may require different survey methodology. For instance, sampling a reef with a benthic grab would be destructive and thus not appropriate, but assessing biodiversity across reefs and sedimentary habitats is. This also applies to different species found in the same space, but which may be surveyed better using different gears, such as those larger dwelling on the seabed or smaller living within the sediment. The only way this can be done currently, and at large scale to determine marine benthic biodiversity hotspots for conservation is by drawing on diverse data sources which may include a combination of survey gears. However, we appreciate that such differences in survey methodology could confound diversity patterns. We therefore provide supplementary results to test our hypotheses when using only the BM data which were all collected with benthic grabs in soft sediments. BM and OBIS data were combined and all transformed to incidence data to produce the largest UK marine benthic dataset the authors are aware of, consisting of 3,626 benthic species from 46,799 multi‐species observations. The OBIS data we used did not share any duplicate observations with the BM data. Where we integrated data, we used species incidence information which is not susceptible to issues associated with duplicate observations (duplicated species incidence records still give a value of 1; see Figure S1 for spatial and temporal distributions across datasets). We focussed on the period 1985–2016, where complementary covariate data exist (see below), and considered only records identified to species.

### 2.2 Biodiversity estimation

We recognize that because the datasets have unique properties (e.g. different spatial and temporal distribution of observations; Figure S1), we cannot make meaningful comparisons between hotspot locations. However, we look for similarities in β‐diversity patterns across data to reveal whether evidence for distinct communities is common to both while also using the combined dataset to provide the most spatially and temporally comprehensive assessment possible across all of the diversity metrics. Specifically, BM count data enable estimates to be based on rarefaction from local to regional scales, whereas incidence data (i.e. OBIS and BM combined) have higher spatial and temporal coverage than the BM data alone, but only γ‐diversity estimates are based on rarefaction (i.e. where multiple cells enable sample‐based rarefaction, whereas α‐ or β‐diversity use observed values).

We anticipated that variation in sample size (e.g. individuals encountered), in the number of observations per site (primarily affecting α‐diversity), in the number of areas surveyed (primarily affecting γ‐diversity), and in the sum of the distances between surveyed areas (i.e. larger areas compared to smaller areas) could affect diversity estimates. And because β‐diversity here is estimated using α‐ and γ‐diversity (see below), it is likely susceptible to variation in all these potential survey effects. We therefore devised a spatially gridded sampling structure to perform random sub‐sampling, thereby enabling us to control the number of multi‐species observations assessed within a given area. Data were nested in 5 km grid cells (henceforth, ‘sample cells’) using latitude and longitude, together with month and year information, which identified whether multiple unique local multi‐species observations existed per sample cell for any given time. Furthermore, we include the number of cells and the sum of the distances between them as covariates in a Random Forest analysis (see below) to reveal whether survey effort was affecting diversity estimates irrespective of whether they were based on rarefaction.

Estimates of α‐diversity were always made at the level of unique multi‐species observations and based on either observed species or rarefaction where we use incidence or count data, respectively. Unique multi‐species observations based on species incidence were used to estimate γ‐diversity within a 25 km radius of each sample cell using rarefaction for all datasets (analogous to regional richness). We only considered areas where there were at least three sample cells within a radius of 25 km required to estimate β‐ and γ‐diversity so that the distribution of our diversity estimates correspond exactly. Estimates based on rarefaction were made using the R package *iNEXT* (Hsieh et al., 2014) and were considered independently to those based on observed values in our analyses. β‐diversity was calculated as:

(1) $$\beta =\gamma /\bar{\alpha },$$

where $\bar{\alpha }$ represents mean α‐diversity (Jost, 2007; Tuomisto, 2010). β‐diversity is thus an estimate of the effective number of communities in a given area and can be expressed on a relative scale as:

(2) $$\beta =\gamma ‐\bar{\alpha }/\left(N‐1\right)\bar{\alpha },$$

where *N* represents the number of sample cells (henceforth referred to as turnover). We also looked at pairwise turnover (Simpson dissimilarity, after Baselga, 2010) between cells within a 25 km radius to reveal whether there were sharp changes in assemblage structure between nearby cells that may be missed using 25 km turnover which uses mean α‐diversity. Whether 25 km or pairwise, we should expect turnover to have a bimodal distribution with peaks close to either extreme if superorganism structure is prevalent (i.e. showing very similar and/ or very dissimilar local site differences), but more intermediate values would suggest that individualistic processes prevail. Pairwise dissimilarity was estimated in R using the *betapart* package (Baselga & Orme, 2012).

While the scales of assessment are somewhat arbitrary, our aim was to assess whether decomposing diversity into its component parts could tell us more than the individual metrics on their own while controlling for variation in survey effort. To do this, we needed to look across spatial scales and across datasets in a systematic way. These scales could be readily adjusted for specific management purposes. This proved essential as, even after rarefaction and extrapolation, diversity estimates were primarily driven by survey effects (Figures S2 and S3). Hence, we standardized effort using random sub‐sampling across all analyses presented in the results such that γ‐ and β‐diversity estimates were based on only ten unique multi‐species observations and α‐diversity on a single multi‐species observation (see Figure S4 for a schematic of this design). We retain the sum of the distances in our models using the sub‐sampled data to show whether sample clustering was still an important factor affecting our diversity estimates.

### 2.3 Covariate data collation

Using the same spatial grid as for diversity estimation, we collated environmental variables considered important determinants of benthic assemblage structure (e.g. Barrio Froján et al., 2012; Cooper & Barry, 2017; Couce, Engelhard, et al., 2020; Davies et al., 2004; Renaud et al., 2009), including depth, sediment composition, bed shear stress, temperature, salinity, nitrate, phosphate, dissolved oxygen, chlorophyll, bottom current speed and particulate organic carbon. We also included data on fishing pressure (hours fishing per year) derived from an analysis of Vessel Monitoring System (VMS) data for UK vessels larger than 15 m and segregated by gear type. This was only available for the period 2009–2016, since VMS usage was not widespread before that; we approximated the average of those 7 years to the entire study period. We acknowledge that fishing effort changes over time, however such changes tend to be slow and gradual due to fishing vessels being based at particular ports, having traditional fishing grounds and fishing preferences, and having quotas associated with particular areas (e.g. see Couce, Schratzberger, et al., 2020). For the 7 years with data, we found a strong correlation between the annual values and the average of the 7 years (see Figure S5). For the full list of spatial gridded environmental and anthropogenic pressure covariates and their sources, see Table 1. For each of these gridded covariates, we also looked at the mean of pairwise differences between surveyed sample cells, which provides a measure of their heterogeneity (e.g. depth difference between cells). The labels referring to these pairwise differences have the suffix ‘*df*’ added to the variable name (variable names are listed in Table 1).

**TABLE 1:** Gridded environmental and anthropogenic pressure datasets used in the study, their characteristics (units, time period covered and spatial resolution) and sources. The label assigned to the variable in our study is indicated in the first column in parentheses, or when not provided it matches the variable name. Variables in our results referring to pairwise differences have the suffix ‘*df*’ added to the variable name

Covariate and variable name	Unit	Time coverage	Resolution	Data source
Depth	m		30 s	General Bathymetric Chart of the Oceans (GEBCO; www.gebco.net)
Temperature	K	1985–2013	⅟₉° longitude by ⅟₁₅° latitude	Modelled with version 3.4 of the Nucleus for European Modelling of the Ocean (NEMO) ocean model (Madec, 2008) coupled to the European Regional Seas Ecosystem Model (ERSEM; Butenschön et al., 2016). Datasets available from http://marine.copernicus.eu/; datasets ‘NORTHWESTSHELF_ REANALYSIS_PHYS_004_009’ and ‘NORTHWESTSHELF_REANALYSIS_BIO_004_011’.
Salinity	e^−3^ (N/A)			
Nitrate concentration (‘*Nitrate mean’* or ‘*log10(nitrate)’*)	mmol/m^3^			
Phosphate concentration (‘*Phosphate mean’* or ‘*log10(phosphate)’*)	mmol/m^3^			
Dissolved oxygen (‘*Dissolved oxygen mean’* or ‘*log10(DO)’*)	mmol/m^3^			
Current speed (‘*Bottom current’*)	m/s			
Chlorophyll‐*a* concentration (‘*Chlorophyll’*)	mg/m^3^	1998–2016	4 km	Ocean Colour Climate Change Initiative dataset, Version 3.1, European Space Agency, available online at http://www.esa‐oceancolour‐cci.org.
Sediment fraction of: Sand Mud Gravel	Proportion (0–1)	N/A	7.5 arc seconds	Dataset described by Mitchell et al. (2019).
Particulate organic carbon (*POC*)	%	N/A	500 m	Dataset described by Diesing et al. (2017).
Shear stress (‘*Shear stress’* for mean of the 7 years, *‘Shear stress SD’* for standard deviation)	m/s	1975–1981	0.125°	Modelled by GETM (Burchard & Bolding, 2002), hindcast model run described in Bricheno et al. (2015).
Fishing pressure for: Beam trawls Otter trawls Pelagic trawls	Hours per year	2009–2016	0.05°	Analysis of Fisheries Administrations’ VMS data provided by the MMO, for UK vessels larger than 15 m.

In addition to the gridded covariate data, we included the mean annual index values for the Atlantic Multidecadal Oscillation (AMO) and the North Atlantic Oscillation (NAO). These two indices track large‐scale environmental change associated with contrasting precipitation and warm and cool climate phases, the AMO being of lower frequency (60–80 years) relative to the NAO (<30 years; Enfield et al., 2001; Hurrell, 1995; Jones et al., 1997). Both indexes have been linked with changes in marine plankton, benthic invertebrates and fish assemblages, through to marine ecosystem‐level change (e.g. Edwards et al., 2013; Nye et al., 2014; Ottersen et al., 2001). We estimate the colonization potential of the wider species pool for each sample cell and each year via sample‐based rarefaction by randomly selecting 50 cells (or at least 30 cells where data from <50 cells was available) from the wider regions beyond 25 km (wider regions are defined in Figure S1 as northwest, northeast, southeast and southwest UK waters). Year, longitude, latitude, distance to the nearest coast and the influence of survey effects that could confound our diversity assessment, namely the number of unique multi‐species observations per cell, the number of cells surveyed within 25 km and the sum of the pairwise distances between cells (i.e. sample clustering), were included in our full list of covariates.

Pairwise Pearson correlation coefficients for all covariates considered were computed to assess multicollinearity between them using data from where we had multi‐species observations in the R package *stats* (R Core Team, 2017). A variable was removed if it correlated with another >0.7, retaining those which represent a direct measure of environmental conditions (e.g. temperature instead of latitude) and those more readily collected (e.g. salinity instead of the difference between salinity across sites; Figures S5 and S6). Where there were few temporal observations and the mean correlated with its temporal counterpart, for example, fishing effort datasets, we used the mean. Any further missing covariate data were imputed using the R package *missForest* (Stekhoven & Bühlmann, 2012).

### 2.4 Biodiversity assessment

For the purpose of our analyses, hotspots are defined here as the 10 most diverse areas ranked using either γ‐, β‐ or α‐diversity. We tested whether hotspot locations were conserved across diversity metrics and spatial scales. Variation in survey distribution over time meant we could not demonstrate if hotspots were temporally conserved (Figure S1). Instead, we tested for the crossing of rarefaction curves over time to reveal the consistency of scale dependence (Chase et al., 2018). Consistently, crossing rarefaction curves indicate that the ranking of sites by richness re‐orders as the spatial scale considered increases. This would undermine the concept of defining hotspots using one metric and/ or one spatial scale.

Random Forest analysis (Breiman, 2001) was used to understand the relationships between diversity metrics and covariates because of their ability to detect nonlinear responses and interaction effects between variables (e.g. Lawler et al., 2006). Regression trees recursively split the dataset into the most homogeneous groups possible in relation to the response variable. Random Forests are a collection of regression trees trained on bootstrap samples of the training set, with the model selecting a randomly chosen subset of the covariates to determine each split of a tree. Due to spatial and temporal clustering of observations inherent in collated survey data (Figure S1) and potential for autocorrelation between proximal observations in space and time to affect our results, we followed the approach described by Hengl et al. (2018) to explicitly add spatiotemporal predictive variables to the Random Forest analysis. Specifically, we include the sampling year (to account for temporal autocorrelation) and the distance from the sample cell to the centre of every other sample cell in the grid up to a maximum of 100 km (to account for spatial autocorrelation at local to mid‐scales). The models were built in R using the *ranger* package (Wright & Ziegler, 2017).

The relative importance of the covariates in each Random Forest model was based on node impurity, a standard approach based on the reductions of the variance of the response variable each time that a covariate is used for a split. Partial dependence plots were used to show the relationship between the response and a single covariate while keeping the other covariates fixed at their average values (this may not be realistic and lead to misleading results in the case of strongly correlated variables, so these plots should be interpreted with care). We compared our full model results, which included environmental covariates, to those with only survey effects as covariates (i.e. year, latitude, longitude, distance to coast and sum of the distances between surveys) and observed diversity estimates to disentangle the effects of environmental change and survey distribution (i.e. temporal changes in the spatial distribution of observations inherent in collated survey data) on our results. Change in relation to ‘Year’ in our survey effects models represents the diversity we might expect to observe in the absence of survey effects; whereas change in relation to ‘Year’ in our full model reveals any outstanding temporal variation in diversity not captured by our covariates.

## 3 RESULTS

**FIGURE 1:**
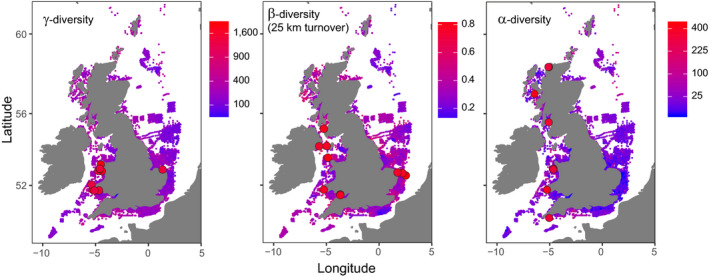
The top 10 marine benthic ‘hotspots’ (large points) plotted over spatial estimates of γ‐, β‐ and α‐diversity across the UK EEZ using the combined dataset. Values are based on means where multiple temporal observations exist

**FIGURE 2:**
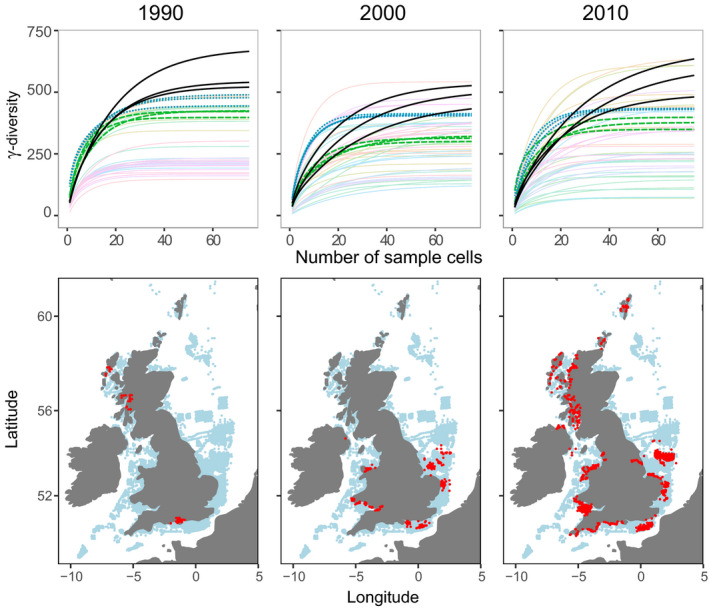
Top panels: rarefaction of marine benthic assemblages used to estimate γ‐diversity for years 1990, 2000 and 2010. Crossing curves demonstrate that the sites rank‐order based on richness is not conserved as the number of sample cells (i.e. spatial units) increases, and this feature is consistent over time (*n* = 50 cells were randomly selected in 2000 and 2010 where there were data for >50). We highlight areas which may have relatively low to intermediate species richness based on a low number of sample cells (black lines) compared with other areas (e.g. blue dotted and green dashed lines) but, because of higher dissimilarity between local assemblages, tend to have some of the highest species richness values at larger scales. Based on these results, we would draw contrasting conclusions about which area was most diverse depending on whether we looked at 1, 20 and 60 sites within a region. Bottom panels: maps show differences in spatial data distribution between respective years (red = data collected in that year, blue = all data)

**FIGURE 3:**
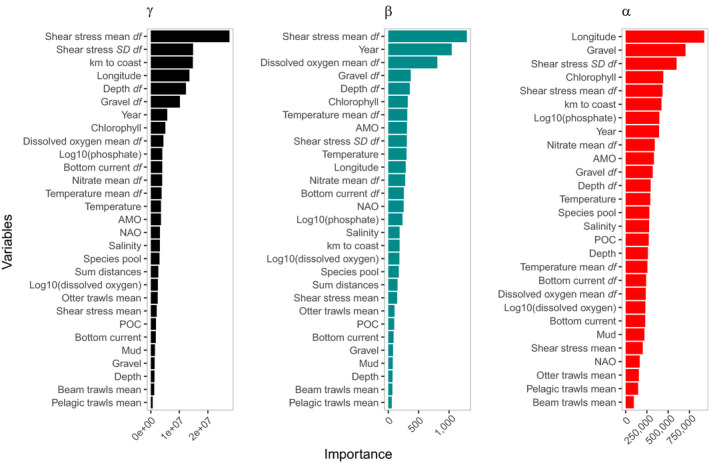
Variable importance based on node impurity ordered along the *y*‐axis from most important (top) to least important (bottom) following Random Forest analysis on the combined data. The suffix ‘*df*’ represents a variable's heterogeneity based on mean pairwise differences across selected sample cells within a 25 km radius

**FIGURE 4:**
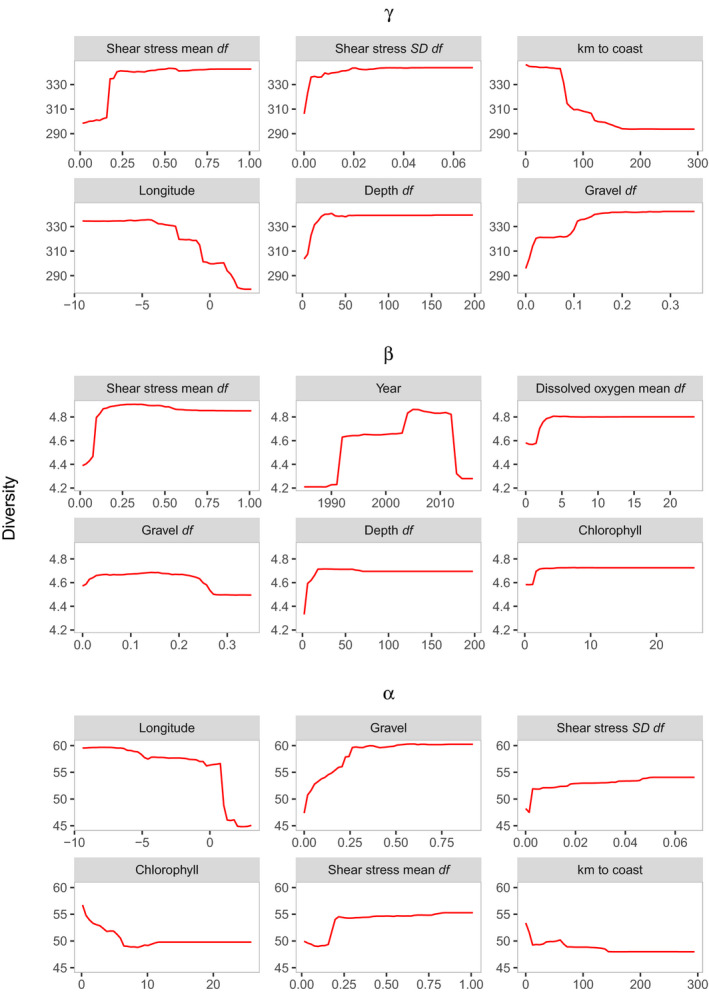
Partial dependence plots showing model predictions (red line) of γ‐, β‐ and α‐diversity (*y*‐axis) using the combined dataset in response to the six most important covariates (*x*‐axis) as determined by node impurity (Figure 3) while keeping other variables fixed at their average values. The suffix ‘*df*’ represents a variable's heterogeneity based on mean pairwise differences across selected sample cells within a 25 km radius

**FIGURE 5:**
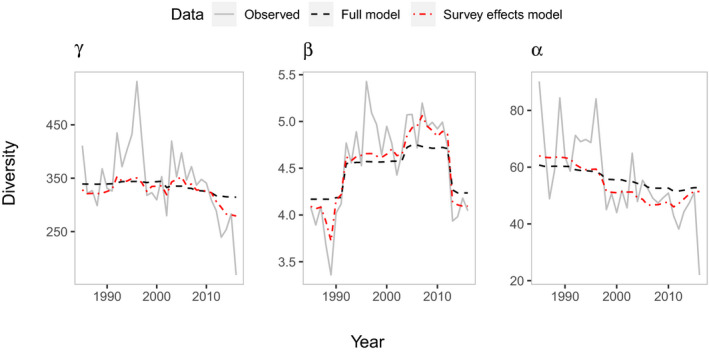
Observed versus partial dependence plots for full model and survey effects model estimates of temporal diversity. Change was partly captured by our environmental and survey covariates, revealed by the more limited change related to ‘Year’ in the full models, followed by the survey effects models, with most variation in our observed values

Hotspots were largely inconsistent across metrics (Figure 1; Figure S7; Table S2) and richness was scale‐dependent over time (Figure 2), in support of our first hypothesis. Using the combined dataset alongside covariates, our random forest models captured 65.6%, 72.2% and 36.8% of the variation (based on out of bag *R*‐squared) in γ‐, β‐ and α‐diversity, respectively. Each diversity metric related uniquely to the covariates, in support of our second hypothesis (Figures 3, 4, 5; Figure S8). Regional factors relating to habitat heterogeneity, including variation in shear stress, depth and substrate, as well as larger‐scale factors such as proximity to coast and longitude (the latter capturing a decreasing species richness gradient moving from the Atlantic to the North Sea species pools), and local factors including substrate, phosphate and chlorophyll concentrations were important predictors of diversity across our models (Figures 3, 4, 5; Figures S9 and S10). This suggests that regional‐ to ecosystem‐scale processes chiefly determined assemblage structure and the distribution of biodiversity hotspots, even at local scales, with small‐scale habitat characteristics, such as substrate playing an important but subordinate role. Chlorophyll, phosphate and differences in dissolved oxygen concentrations ranked the most important gradients associated with anthropogenic pressure (i.e. a potential coastal eutrophication gradient; Figure 4; Figures S9 and S10), the former two relating negatively to α‐ and γ‐diversity, the latter relating positively to β‐diversity. Various trawling pressures were consistently poor predictors in our models.

Comparing output from our full models to our survey effects models and observed values reveals that temporal change in diversity was partly captured by environmental covariates and partly by survey covariates with some remaining unexplained variation (Figure 5). There was more limited change related to ‘Year’ in the full models, followed by the survey effects models, with most variation in our observed values. Estimates based on our survey effects models (i.e. what we might expect to observe in the absence of survey effects) showed declines in γ‐ and α‐diversity of 14.9% and 19.7%, respectively, over the study period, and variation in β‐diversity of 26.5% with little overall difference between 1985 and 2016 estimates.

**FIGURE 6:**
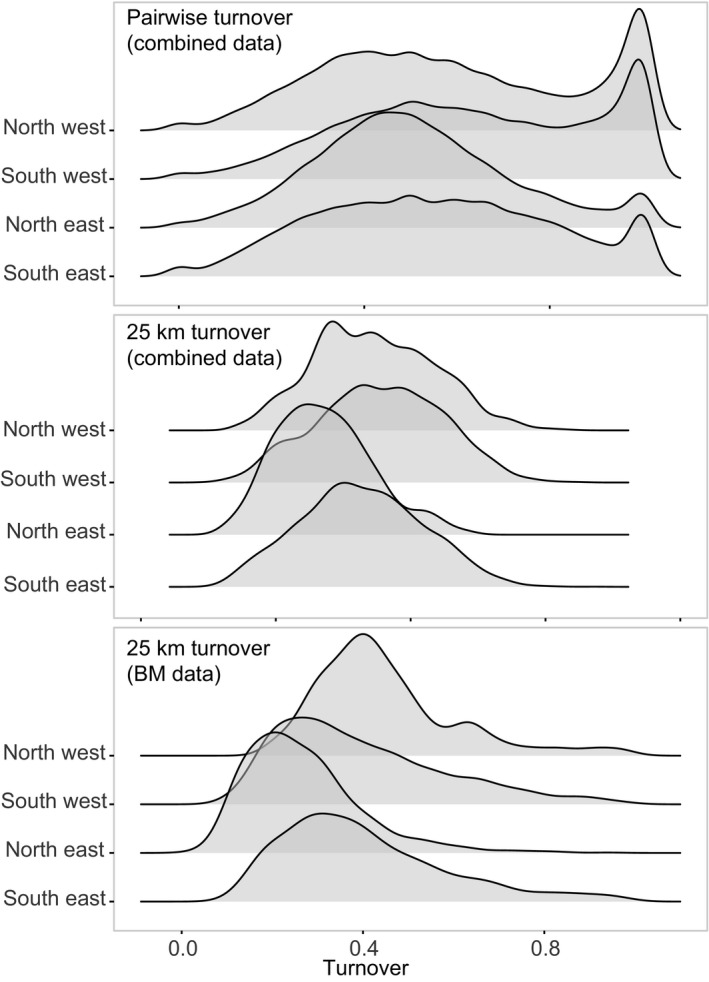
Density plots showing the distribution of pairwise and 25 km turnover estimates (i.e. β‐diversity) between cells sampled within the same year using the combined and BM datasets, respectively. Values were mostly intermediate, rather than bimodally distributed between extremes, indicating that many species were shared between unique multi‐species observations and thus individualistic processes were more prominent in general. Data have been split across UK regions (Figure S1) to show that this pattern was largely conserved through space

We found evidence of both individualistic and organismic assemblage structure with considerable variation in species turnover that was largely consistent across regions and data (i.e. where estimates were based on observed values in the combined incidence data or via rarefaction using BM count data), in support of our third hypothesis (Figure 6). The majority of pairwise and 25 km turnover values were intermediate rather than distributed bimodally between the extremes. Bimodal peaks were evident in pairwise turnover between intermediate and high values demonstrating that almost completely unique assemblages were observed in some nearby cells. However, such a peak was not evident in 25 km turnover suggesting that sharp dissimilarities were outweighed by those more intermediate, and thus individualistic processes were more prominent in general.

## 4 DISCUSSION

The hotspot concept is intuitive, so it is understandable why it has such resonance in biodiversity conservation (Marchese, 2015; Myers et al., 2000). Here, we offer new insights into how such hotspots are generated and how they behave in space and time. The novelty in our study does not lie with the distribution of marine benthic biodiversity hotspots nor the influence of specific covariates per se, but rather the multi‐dimensional perspective afforded using α‐, β‐ and γ‐diversity together, in the scale dependence of the responses to covariates and the ubiquity of gradual change in assemblage structure. These findings are key to informing marine management and conservation because strategies that act only locally, assume clearly delineated assemblages or focus on a single component of diversity risk failing to detect change, identify its direction or disentangle the influence of local from larger‐scale processes, which could undermine effective intervention (Chase et al., 2018, 2019; McGill et al., 2015). Meaningfully incorporating a multi‐dimensional perspective of biodiversity into national and international conservation programmes, monitoring or regulatory frameworks, such as via the joint use of α‐, β‐ and γ‐diversity, is thus timely (Isbell et al., 2017; Purvis et al., 2018).

Differences between the North Sea and Atlantic species pools captured by the longitudinal gradient was an important predictor of α‐ and γ‐diversity (Figures 1 and 4; Figures S7 and S8) which suggests the success of local measures will be contingent on conserving species and their ability to disperse at large scales (Witman et al., 2004), beyond the wider regions we used to assess colonization potential (Figure S1). Using species relatedness rather than patterns in α‐, β‐ and γ‐diversity, Somerfield et al. (2009) showed that marine benthic assemblages were primarily organized by regional to ecosystem‐scale environmental gradients, adding support to our findings. Also, contrasting patterns of change in α‐ and β‐diversity have been found in other benthic marine assemblages to the extent that a ‘hotspot’ can also be a ‘coldspot’ for biodiversity (Price, 2002). Dornelas et al. (2014) and Blowes et al. (2019) found that change in temporal turnover, rather than richness, was the principal driver of biodiversity change in global studies across many taxa and biomes, and this was strongest but most variable in marine ecosystems. We find comparable declines in richness to some marine assemblages contained within those global studies, plus we assess assemblage turnover in space as opposed to time, to test for sharp spatial change between assemblages, so our results are not necessarily inconsistent. The novelty here is that we relate spatial and temporal change in diversity to potentially confounding survey effects and environmental gradients, and explicitly address scale‐dependent relationships to direct future assessments, policymakers and managers to where along these gradients change in assemblage structure can be anticipated.

Temporal declines in α‐diversity can partly be explained by its negative relationship with the AMO (Figure S9). Altered thermal and hydrological regimes between AMO cold‐negative (up until ~1997) and warm‐positive (~1997 until present) phases may be responsible for this relationship affecting, for example, the supply of nutrients, thermal conditions, dispersal and higher‐level food web effects (Edwards et al., 2013; Nye et al., 2014). However, the exact mechanisms are not clear and warrant future investigation, and the AMO had relatively little effect on γ‐ and β‐diversity. Considering the remaining temporal changes in diversity, these were decoupled across metrics with declines in α‐diversity evident first, followed by declines in γ‐diversity, while β‐diversity was increasing between ~1990 and 2010, before sharply decreasing after 2012 with no single clear driver. This suggests that many different pressures may be acting locally such that their heterogeneous but cumulative effects have led to widespread biodiversity change, and/or that other environmental drivers not captured in our covariates were important (e.g. novel pesticides; Van Der Sluijs et al., 2015). Warming from climate change is another such possibility which we cannot rule out because our data do not track its effect on assemblages through time, among other candidate covariates which we discuss below.

The main anthropogenic gradients we considered were nutrient concentrations and fishing effort. Sites with altered substrate characteristics, for example, following dredging, were not considered but such activities need careful management given that the proportion of gravel in sediments and its heterogeneity were important determinants of diversity (see also Cooper & Barry, 2017). Species richness tended to be negatively related to gradients associated with eutrophication which were higher in coastal waters (Figure 4; Figure S9). α‐ and γ‐diversity increased above phosphate concentrations of ~0.61 μM which broadly supports the lower threshold mean (0.45 μM) used by OSPAR to assess eutrophication in UK marine waters (Foden et al., 2011). Considering that riverine nutrient inputs have been in decline following policy changes since the late 1980s (Capuzzo et al., 2017; Foden et al., 2011; Lenhart et al., 2010; Tett et al., 2007), other contaminants, such as pesticides, not included in our covariates, could also be responsible for declines in species richness and warrant future investigation.

Like our study, Couce, Engelhard, et al. (2020) found shear stress to be the primary driver of change across North Sea marine benthic assemblages. However, they showed that beam and otter trawling had pronounced effects with clear thresholds of change on specific benthic genera that were not evident at the assemblage level in our assessment. It has also been shown that species richness can be negatively related to trawling in a meta‐analysis of mostly western Europe and north eastern USA control‐impact studies (Hiddink et al., 2020). There are a few possible and not mutually exclusive reasons for these contrasting results: our data are not directly comparable as they cover different areas; although many taxa may respond to trawling, UK marine benthic diversity may be largely insensitive to recent pressure following centuries of extensive fishing (Barrett et al., 2004; Thurstan et al., 2010); and our trawl data, which are highly spatially resolved but averaged over time, may not be a good indicator of trawling pressure when our observations were made. It would be useful in future therefore to specifically select sites where temporal trawl and survey data correspond and apply the threshold approach after Couce, Engelhard, et al. (2020) to the more extensive dataset presented here. Such an approach could reveal where along anthropogenic gradients such biotic thresholds exist and thus gauge where pressures become unsustainable.

Even after rarefaction, diversity estimates were largely driven by effort before we applied random sub‐sampling. This is because the effective number of assemblages sampled (i.e. β‐diversity) increased with survey effort, affecting the shape of the accumulation curve which systematically increased the asymptote (Figures S2 and S3; see also Whittaker, 1960). Studies which do not explicitly address survey effects through both time and space before applying rarefaction, therefore risk reporting change associated with effort, which can vary dramatically (Figure 2; Figure S1), rather than biodiversity change *per se*. Spatial and temporal change in species richness could therefore be missed or misinterpreted if survey effects are not addressed prior to estimating diversity. We strongly advocate presenting diversity estimates alongside potential survey effects to make this transparent in future assessments, for example, using a spatially structured approach such as the one we present.

Future biodiversity assessments must therefore carefully control the number and distribution of samples, including measures for sample clustering, in both time and space to detect change and reveal its causes. Large‐scale spatial and temporal change in diversity cannot be disentangled if data only exist for a unique area or a unique combination of areas over time, constraining subsequent analyses. We only assess trends at local to regional scales because the substantial annual variation in the spatial distribution of survey effort presents a major challenge to estimating temporal change in marine benthic diversity at the UK scale which we do not attempt to address. However, simply increasing the number of annual observations alone may not improve our understanding of the causes of change. A truly integrated ecosystem monitoring programme that explicitly considers how processes link sampled components would provide important insights into causal drivers of biodiversity change that are not possible using such data collated without an overarching design (Arkema et al., 2006; Kupschus et al., 2016). For this reason, we must stress that our environmental‐diversity modelling results are exploratory and do not conclusively show significant declines in UK marine benthic species richness. Rather, they show noteworthy trends relevant to biodiversity conservation and future monitoring and assessment practices which could, for example, be applied to predict where we might find new areas of high biodiversity by filling in the gaps in Figure 1 where there is currently little or no biological data.

By combining the structured and targeted BM dataset with the unstructured, opportunistic data from OBIS, we aimed to benefit from the strengths inherent in each to derive a more comprehensive picture of benthic biodiversity in space and time than either resources would enable alone. The importance of drawing on multiple sources of data in such biogeographic studies has been emphasized elsewhere (Chollett & Robertson, 2020). We show how some of the limitations in such data collations can be addressed and were able to reveal that the prevailing structuring force of the UK marine benthos was individualistic. Dispersal and variation in interaction strength also play significant roles in reducing β‐diversity (Liautaud et al., 2019). It is therefore rarely clear if nearby observations come from one or multiple assemblages, assemblage continua or some combination of these, and this has profound implications for how an area is assessed and conserved. Our findings do not preclude the use of strata as a pragmatic approach to surveying large areas with finite resources, and in also highlighting areas with unique assemblages whose loss would represent wider regional species loss. Rather, we highlight the importance of retaining information and a degree of flexibility in future monitoring designs useful for biodiversity assessments that are not beholden to strict delineations between areas that may not exist (Dickey‐Collas, 2014; Kupschus et al., 2016; Murdoch et al., 2014). Without assuming clear delineations between areas, joint use of α‐, β‐ and γ‐diversity provides a multidimensional perspective of biodiversity which is key to its assessment (Chase et al., 2019; Isbell et al., 2017), revealing how a given area will respond to environmental change and the appropriate scale for management to be effective, for example. We hope insights from this study inform both the design of future monitoring programmes and conservation policy so that anthropogenic pressures on biodiversity are better assessed, understood and mitigated.

## AUTHOR CONTRIBUTION

M.S.A.T., E.C., M.S. and T.J.W. conceived the ideas and designed the methodology; M.S.A.T., E.C., T.J.W., K.M.C. and M.G. collated the data; M.S.A.T., E.C., M.S., T.J.W. and K.M.C. analysed the data; M.S.A.T. led the writing of the manuscript. All authors contributed critically to the drafts and gave final approval for publication.

## Supporting information

Supplementary Material

## Data Availability

The data that support the findings of this study are openly available from the Cefas DataHub at https://doi.org/10.14466/cefasdatahub.34 and OBIS at https://doi.org/10.25607/obis.export.dda4657a.
