# Supplementary material for: What’s hot and what’s not: Making sense of biodiversity ‘hotspots’
Source: Glob Chang Biol. 2020 Nov 26;27(3):521–35. doi: 10.1111/gcb.15443 (PMC7839497; doi:10.1111/gcb.15443)
Supplement: Supplementary file 1 — Supplementary Material [file GCB-27-521-s001.docx]

**Supporting Material**

**Title**: What’s hot and what’s not: making sense of biodiversity ‘hotspots’

**Running title:** Making sense of biodiversity hotspots

**Authors**: Murray S. A. Thompson^1^, Elena Couce^1^, Thomas J. Webb^2^, Miriam Grace^2^, Keith M. Cooper^1^ and Michaela Schratzberger^1^

Correspondence: [murray.thompson@cefas.co.uk](mailto:murray.thompson@cefas.co.uk)


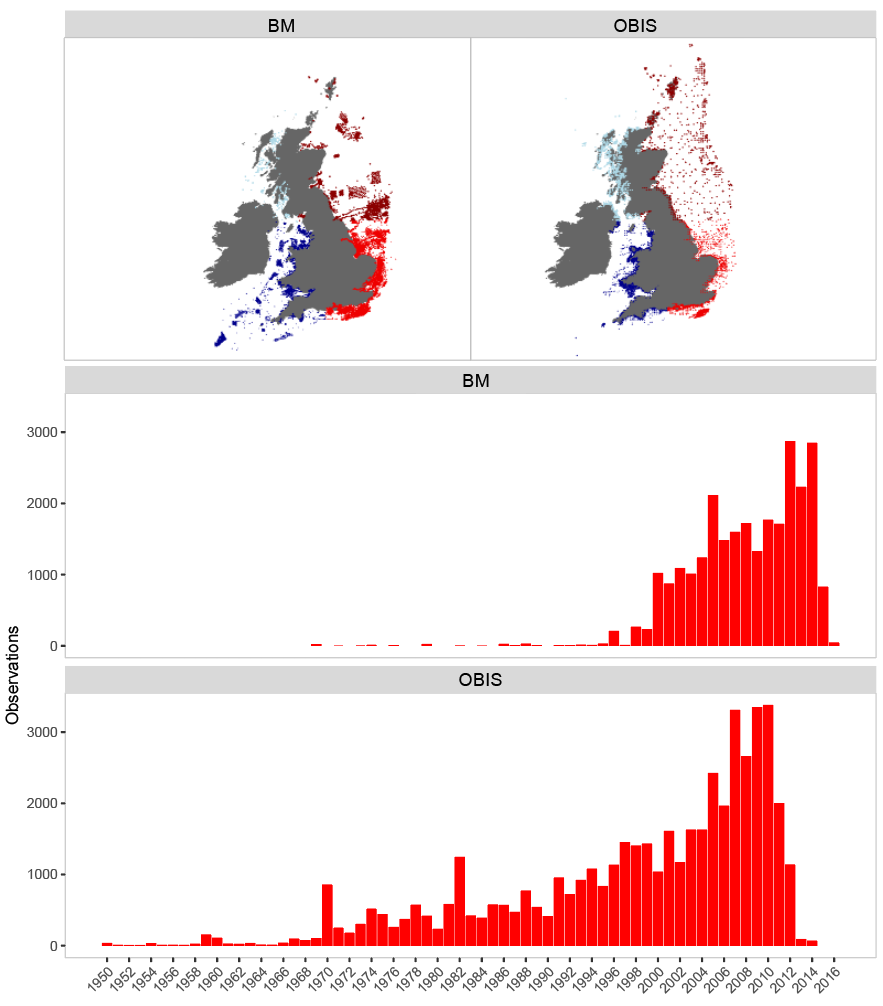


Fig S1. Differences in the spatial and temporal distribution of observations between datasets. UK regions used to estimate species pools for our models and spatially distinguish ß-diversity estimates in Fig. 6 are colour coded in the maps (dark red = north east; red = south east; dark blue = south west; light blue = north west).

Table S1. Phylum occurrence across BM and OBIS data.

| **Phylum** | **BM** | **OBIS** |
| --- | --- | --- |
| Annelida | 369950 | 187126 |
| Arthropoda | 127971 | 91007 |
| Brachiopoda | 15 | 191 |
| Bryozoa | 1580 | 32467 |
| Cephalorhyncha | 100 | 465 |
| Chordata | 7170 | 27358 |
| Ciliophora | 1306 | 28 |
| Cnidaria | 5306 | 39140 |
| Echinodermata | 33234 | 50536 |
| Foraminifera | 108 | 212 |
| Hemichordata | 24 | 30 |
| Mollusca | 115390 | 143172 |
| Nematoda | 1 | 4523 |
| Nemertea | 1791 | 943 |
| Phoronida | 581 | 1105 |
| Platyhelminthes | 62 | 627 |
| Sipuncula | 639 | 3208 |
| Chaetognatha | 0 | 38 |
| Entoprocta | 0 | 101 |
| Porifera | 0 | 39671 |
| Xenacoelomorpha | 0 | 1 |


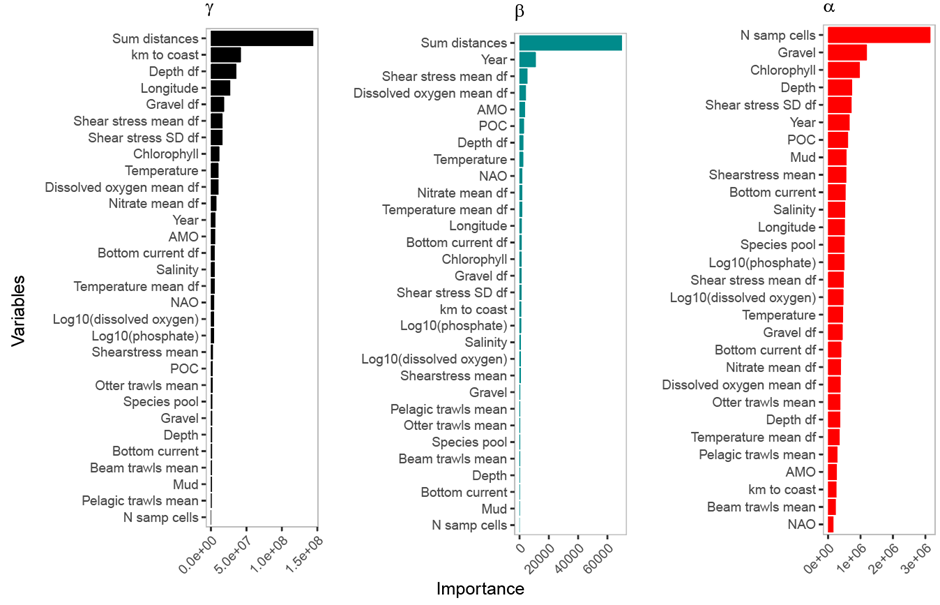


Fig S2. Variable importance for diversity estimates following Random Forest Analysis using the combined dataset uncorrected for variation in survey effort. Values are based on node impurity, ordered along the y-axis from most important (top) to least important (bottom). The suffix “df” represents a variable’s heterogeneity based on mean pairwise differences across selected sample-cells within a 25 km radius.


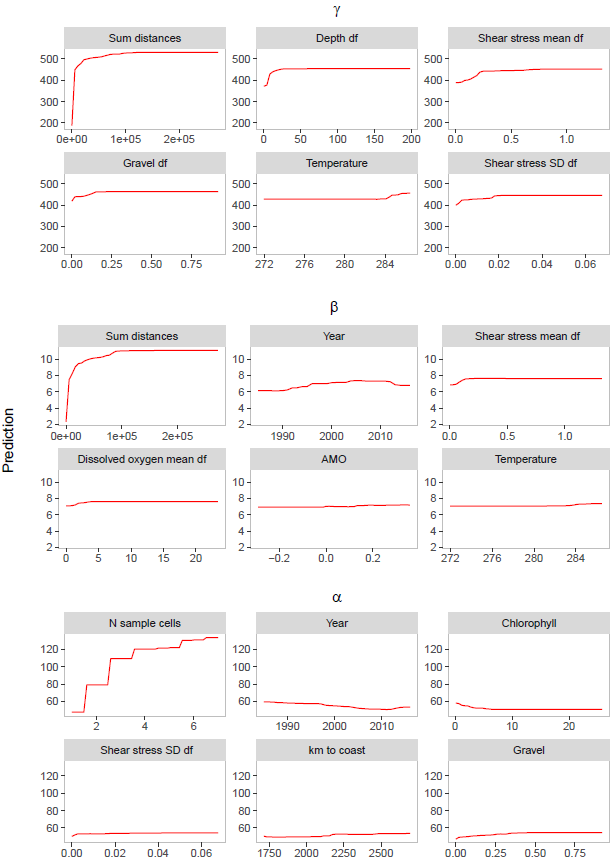


Fig S3. Partial dependence plots showing model predictions of diversity using the combined dataset uncorrected for variation in survey effort in response to the six most important covariates as determined by node impurity (Fig S2), while keeping other variables fixed at their average values. The suffix “df” represents a variable’s heterogeneity based on mean pairwise differences across selected sample-cells within a 25 km radius.


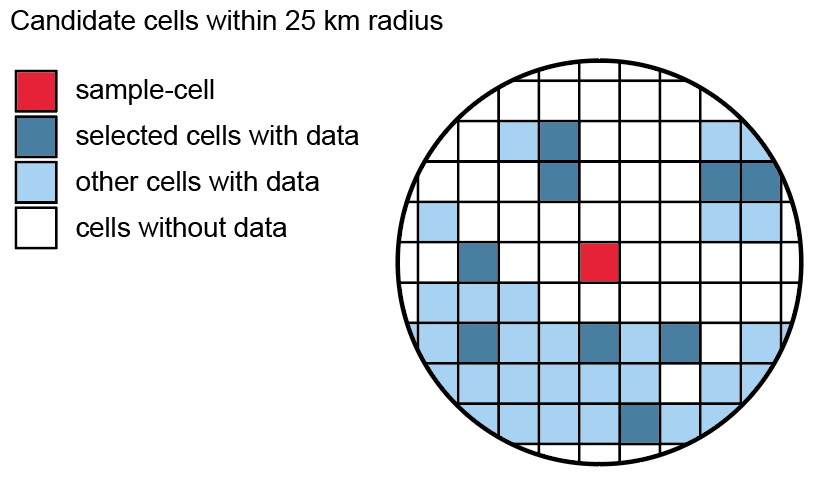


Fig. S4. A schematic of our random sub-sampling design to remove the effect of comparing larger areas to smaller areas. Where there were more than 9 other cells which contained multi-species observations within a 25 km radius from the centre of the sample-cell, we randomly selected 9 cells from those with data. γ- and ß-diversity estimates were based on only ten unique multi-species observations and α-diversity on a single multi-species observation. Areas with <10 cells were excluded from analyses using sub-sampled data.


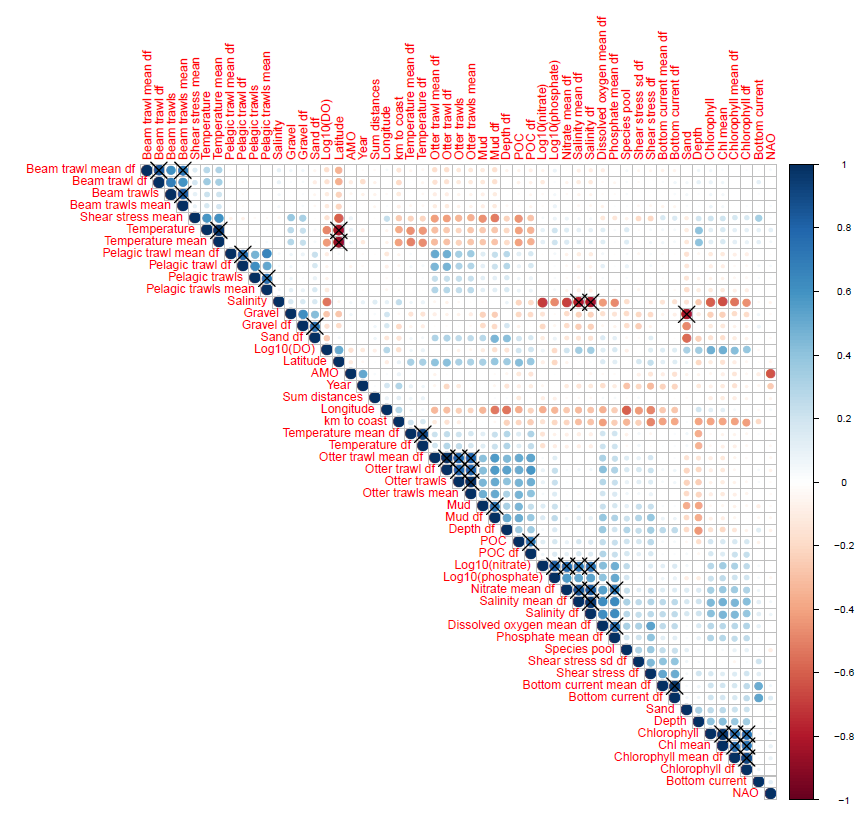


Fig S5. Pairwise Pearson correlation coefficients for all covariates considered were evaluated based on where we had multi-species observations. Black crosses highlight correlations >0.7 where we chose to exclude one of the covarying variables. The suffix “df” represents a variable’s heterogeneity based on mean pairwise differences across selected sample-cells within a 25 km radius.


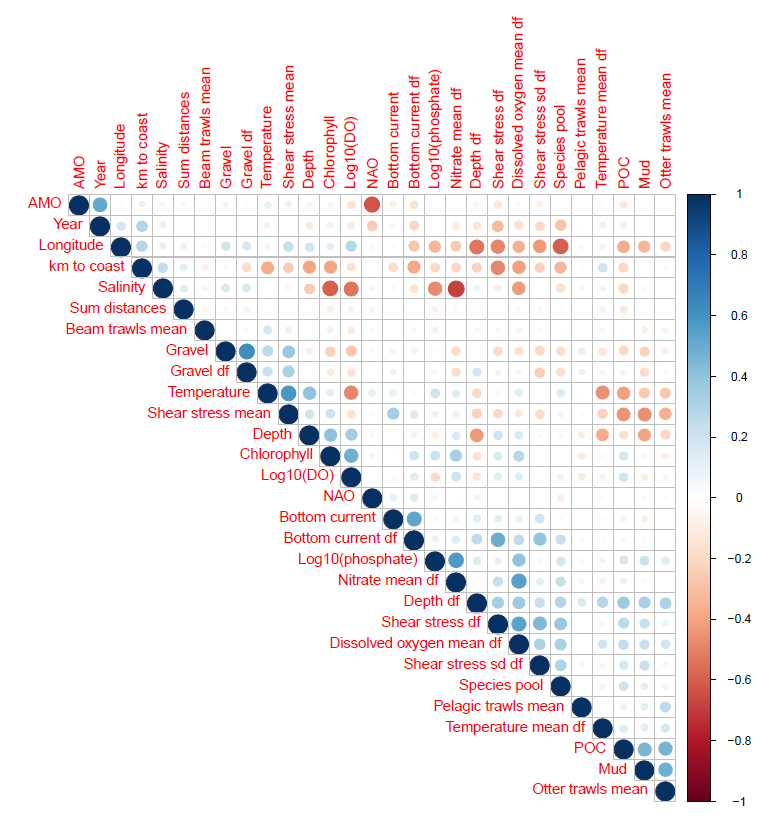


Fig S6. Pairwise Pearson correlation coefficients for only the covariates used in our models. The suffix “df” represents a variable’s heterogeneity based on mean pairwise differences across selected sample-cells within a 25 km radius.

Table S2. The ten most diverse benthic hotspot locations ranked using γ-, ß- (25 km turnover) and α-diversity (observed species) based on means across time using the combined dataset (bold denotes hotspots for each diversity metric).

| Longitude | Latitude | γ | ß | α |
| --- | --- | --- | --- | --- |
| -5.25681 | 51.73511 | **1996** | **0.766** | **462** |
| -4.41323 | 52.82574 | **1817** | 0.622 | 220 |
| -5.1047 | 51.70397 | **1326** | 0.634 | 148 |
| 1.349208 | 52.91957 | **1425** | 0.483 | 44 |
| -4.58766 | 52.96318 | **1448** | 0.6 | 42 |
| -5.38338 | 52.0496 | **1363** | 0.652 | 40 |
| -4.50397 | 53.19585 | **1908** | 0.639 | 37 |
| -4.58832 | 52.94714 | **1495** | 0.652 | 27 |
| -4.74087 | 52.85312 | **1562** | 0.653 | 19 |
| -4.68152 | 51.70791 | **1581** | 0.743 | 18 |
| -5.68393 | 54.21586 | 411 | **0.764** | 43 |
| -3.67785 | 51.44004 | 411 | **0.809** | 33 |
| -3.67609 | 51.41583 | 294 | **0.788** | 22 |
| -4.86152 | 53.57504 | 751 | **0.818** | 11 |
| 2.529333 | 52.56274 | 651 | **0.841** | 6 |
| -5.2327 | 55.1727 | 615 | **0.794** | 6 |
| 2.222926 | 52.7007 | 413 | **0.78** | 6 |
| -4.97067 | 54.23458 | 796 | **0.783** | 3 |
| 1.751567 | 52.73025 | 276 | **0.769** | 2 |
| -6.44386 | 57.04058 | 586 | 0.396 | **419** |
| -5.25402 | 51.73511 | 603 | 0.498 | **402** |
| -5.07545 | 58.40126 | 615 | 0.32 | **389** |
| -4.68747 | 52.9287 | 868 | 0.566 | **382** |
| -4.63261 | 52.9651 | 822 | 0.505 | **355** |
| -5.08519 | 55.5382 | 270 | 0.684 | **350** |
| -5.04706 | 50.04739 | 638 | 0.551 | **333** |
| -5.11279 | 58.40892 | 580 | 0.355 | **330** |
| -4.65519 | 52.91684 | 732 | 0.505 | **326** |


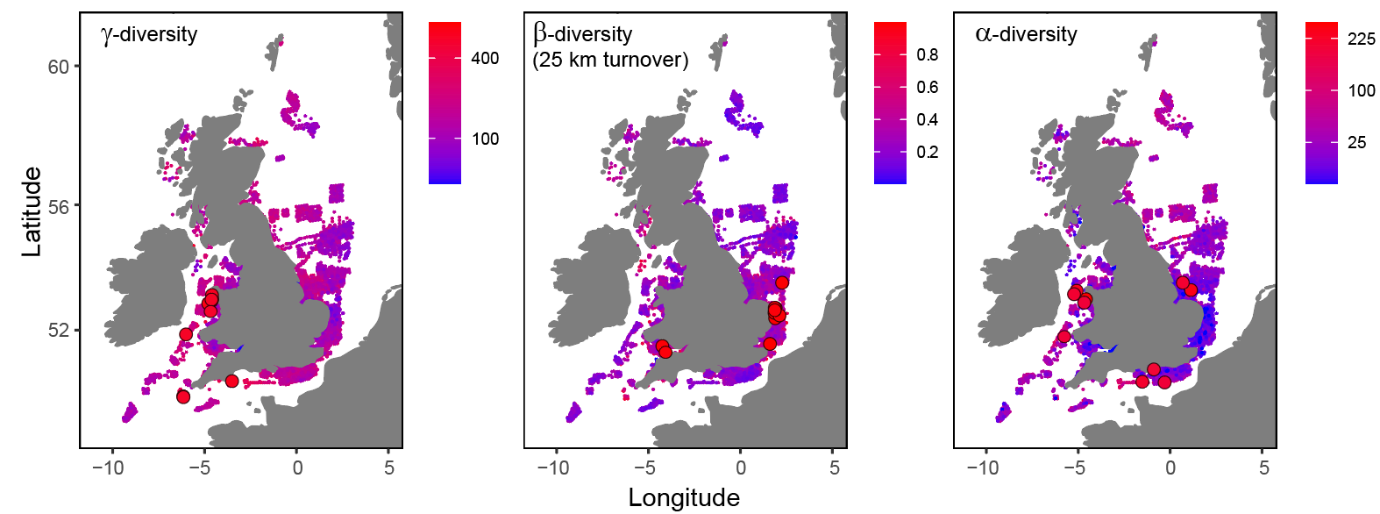


Fig. S7. The top ten marine benthic ‘hotspots’ (large points) plotted over spatial estimates of γ-, ß- and α-diversity across the UK EEZ using the BM data. Values are based on means where multiple temporal observations exist.
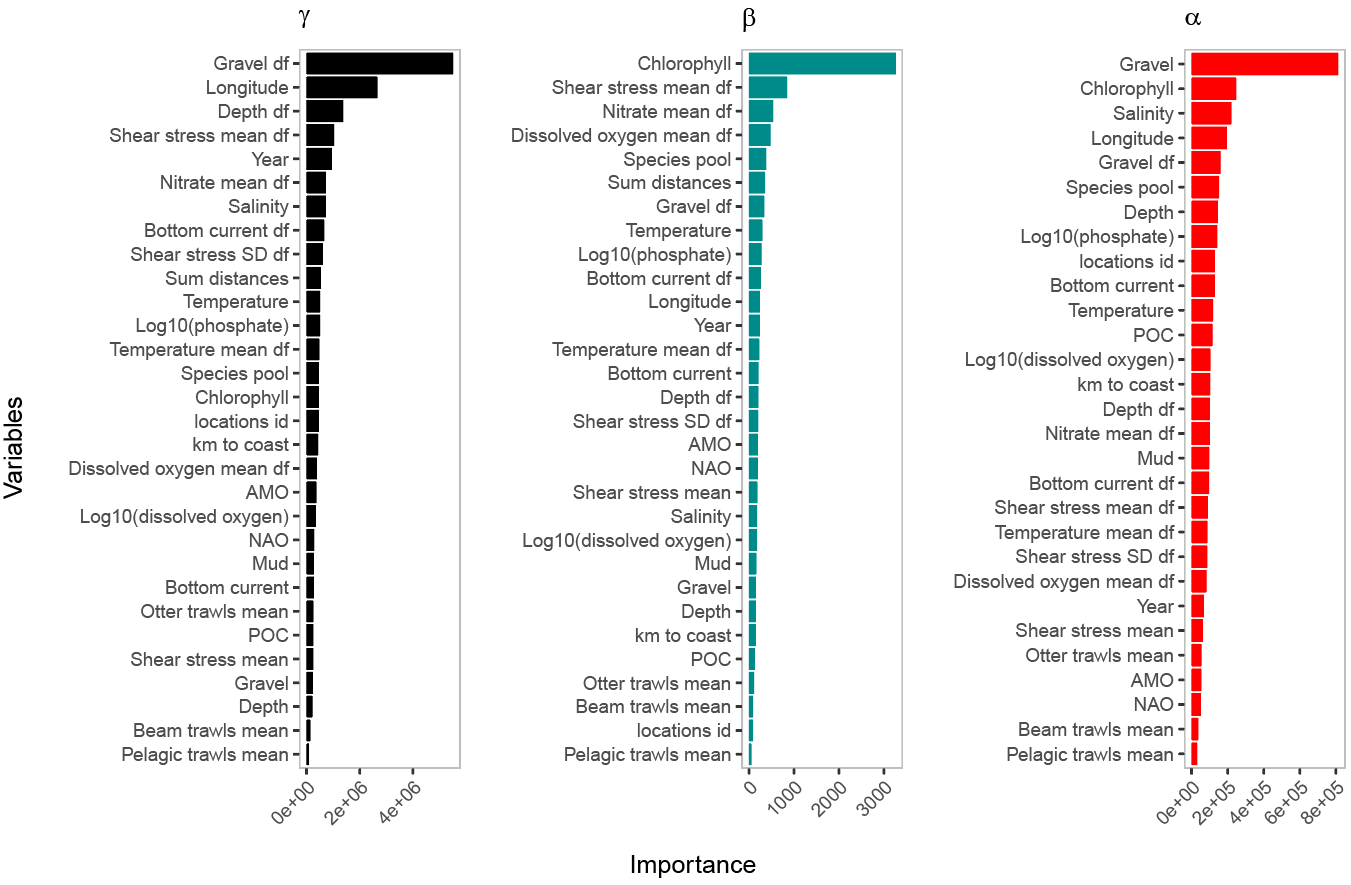


Fig. S8. Variable importance based on node impurity ordered along the y-axis from most important (top) to least important (bottom) following random forest analysis on BM data. The suffix “df” represents a variable’s heterogeneity based on mean pairwise differences across selected sample-cells within a 25 km radius.


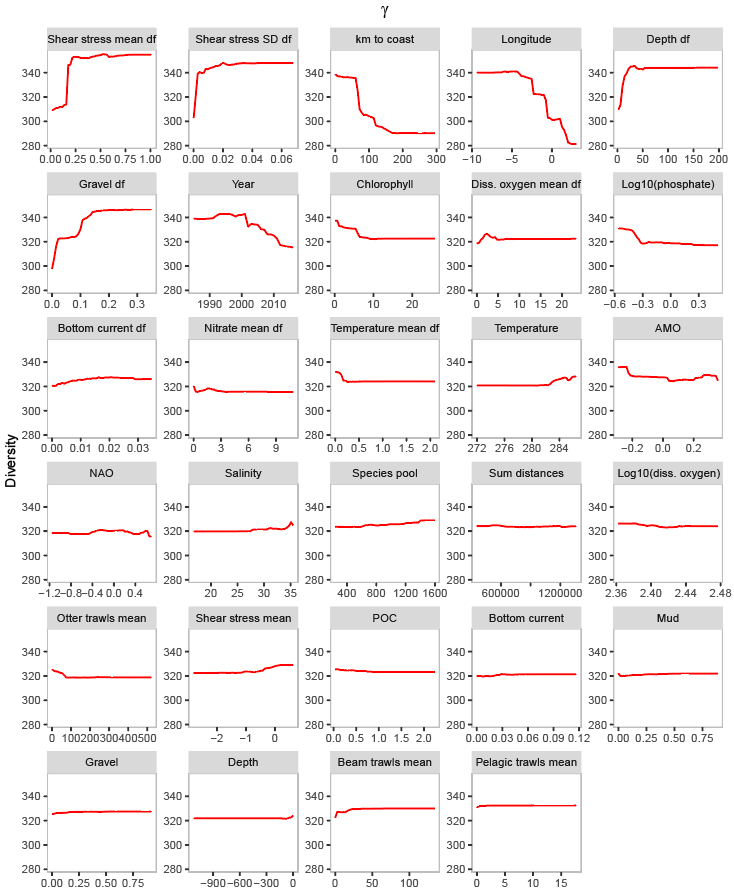


Fig S9. Partial dependence plots showing model predictions of γ-, ß- and α-diversity in relation to all covariates using the combined dataset after sub-sampling, while keeping other variables fixed at their average values. Plots are ordered from the most important covariates (top left) to the least (bottom right) as determined by node impurity.


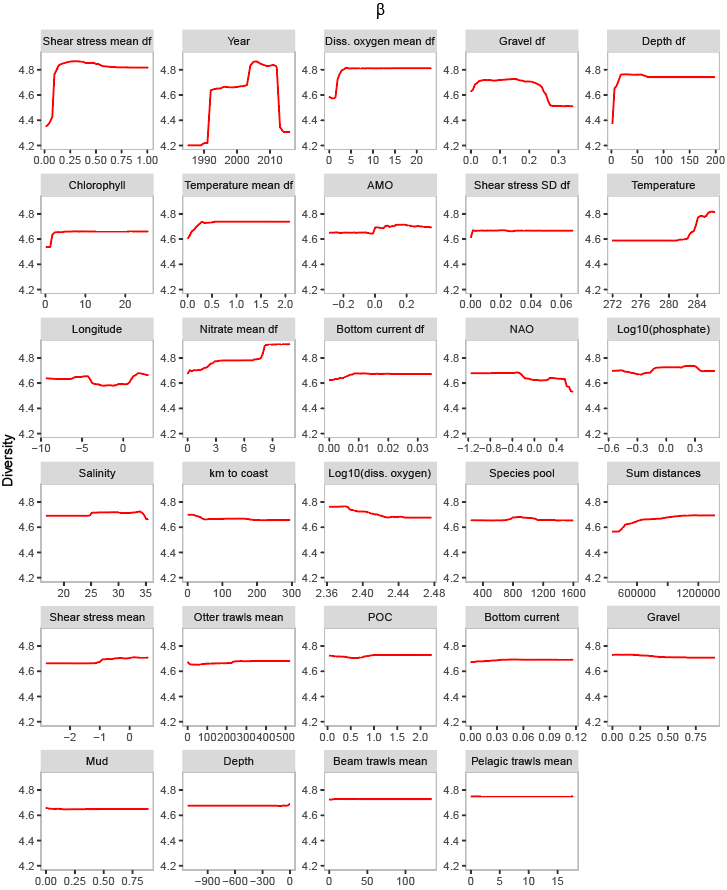


Fig S9 continued.


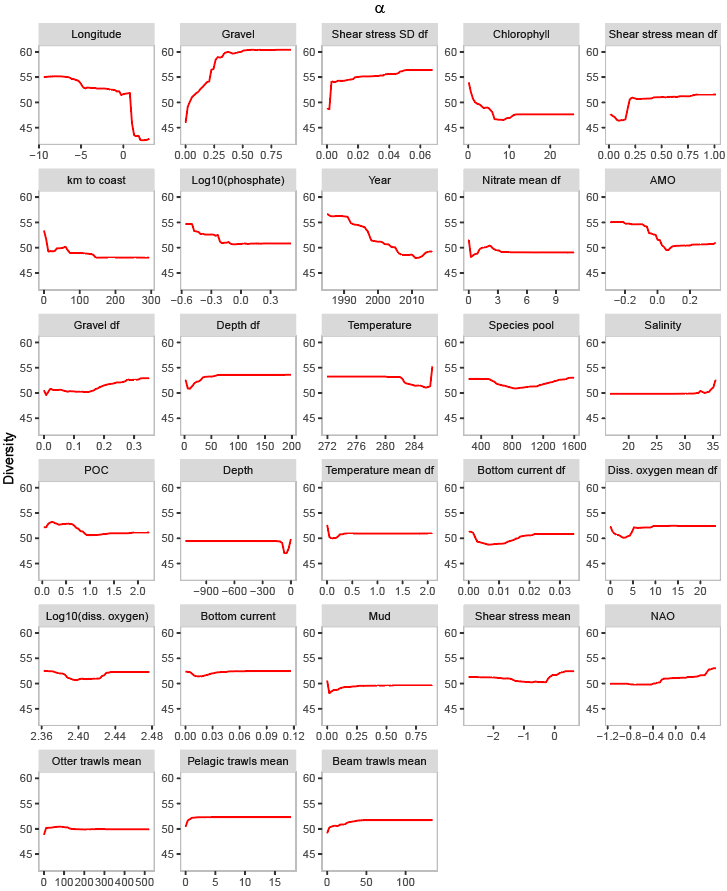


Fig S9 continued.


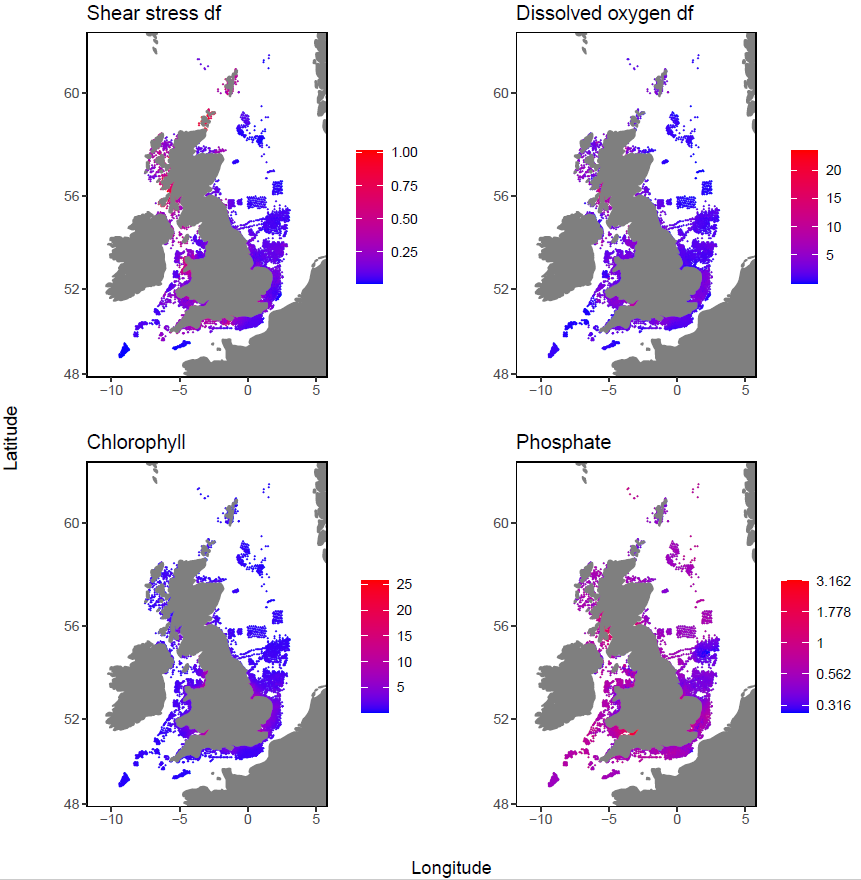


Fig. S10. Corresponding variation in pairwise sample-cell shear stress and dissolved oxygen concentrations, and chlorophyll and phosphate concentrations for our diversity estimates between 1985-2016. We plot average values where multiple observations exist.
